# Nature of back slopping kombucha fermentation process: insights from the microbial succession, metabolites composition changes and their correlations

**DOI:** 10.3389/fmicb.2024.1433127

**Published:** 2024-08-21

**Authors:** Ting Liao, Xiang-Ru Li, Li Fan, Bo Zhang, Wei-Ming Zheng, Jia-Jia Hua, Li Li, Norlia Mahror, Lai-Hoong Cheng

**Affiliations:** ^1^College of Tea and Food Science, Wuyi University, Wuyishan, China; ^2^Food Technology Division, School of Industrial Technology, Universiti Sains Malaysia, Penang, Malaysia

**Keywords:** clean fermentation, symbiosis, kombucha, microbial identification, metabolome

## Abstract

Kombucha, a fermented tea prepared with a symbiotic culture of bacteria and yeast (SCOBY), offers a unique and unpredictable home-brewed fermentation process. Therefore, the need for a controlled kombucha fermentation process has become evident, which requiring a thorough understanding of the microbial composition and its relationship with the metabolites produced. In this study, we investigated the dynamics of microbial communities and metabolites over a 12-day fermentation period of a conventional kombucha-making process. Our findings revealed similarities between the microbial communities in the early (0–2 days) and late (10–12 days) fermentation periods, supporting the principle of back-slopping fermentation. Untargeted metabolite analysis unveiled the presence of harmful biogenic amines in the produced kombucha, with concentrations increasing progressively throughout fermentation, albeit showing relatively lower abundance on days 8 and 12. Additionally, a contrasting trend between ethanol and caffeine content was observed. Canonical correspondence analysis highlighted strong positive correlations between specific bacterial/yeast strains and identified metabolites. In conclusion, our study sheds light on the microbial and metabolite dynamics of kombucha fermentation, emphasizing the importance of microbial control and quality assurance measures in the production process.

## Introduction

Kombucha, a mildly sweet and sour fermented tea beverage, traces its origins back to ancient times, reportedly originating in northeastern China during the Tsin Dynasty around 200 B.C. (Coelho et al., 2020). In recent years, kombucha has experienced rapid market growth, with the fastest expansion rate among all functional beverages (Kapp and Sumner, 2019; Laureys et al., 2020). Kombucha global market value reached USD 2.59 billion in 2021 and is expected to grow with a projected Compound Annual Growth Rate (CAGR) of 15.7% in the forecast period between 2023 and 2030 (Wang et al., 2022a; SkyQuest, 2024). This could be due to its health benefits on reducing cholesterol, blood pressure, kidney calcification, and enhancements to liver, glandular, stomach, and immune system functions (Laureys et al., 2020).

Kombucha is prepared by inoculating a symbiotic culture of bacteria and yeasts (SCOBY) into a sweetened tea broth. Commonly, black tea broth is used as a substrate for fermentation, but variations prepared with other infusions, such as green tea, red tea, oolong tea, and others have become more and more popular. Home-brewed kombucha employs a back slopping technique, where a portion of previously brewed kombucha will serve as a liquid starter culture to initiate new batch of kombucha fermentation (Jayabalan et al., 2014; Miranda et al., 2022). The process may occur at room temperature over 8–15 days, with or without a characteristic floating cellulose biofilm form on the surface of the fermentation broth over time (Roby et al., 2020; Xu et al., 2022).

The advantages of back slopping in kombucha fermentation are numerous. It ensures a consistent microbial population, which is crucial for the stability and quality of the final product. This technique promotes a rapid start to the fermentation process due to the presence of an active, established microbial community, leading to shorter fermentation times and reduced risk of contamination by undesirable microbes. Additionally, back slopping helps maintain the unique flavor profile and health benefits of kombucha by preserving the balance of acetic acid bacteria and yeast species that have adapted to the specific conditions of previous fermentation (Andreson et al., 2023).

The method specificity of back slopping lies in the careful selection and maintenance of SCOBY, because the initial microbial composition and microbial community succession during fermentation can inform stringent hygiene and sanitary practices in kombucha production, as well as the quality of kombucha produced (Grassi et al., 2022). In view of the limited information derived from culture-dependent methods (Marsh et al., 2014; Chakravorty et al., 2016), culture-independent methods have receiving increasing attention in identifying the microbial communities of kombucha in the past two decades. With metagenomic analysis, Kaashyap et al. (2021) reported 34 genera with 200 microbial species identified in commercial kombucha products. The popular bacteria species are belong to the genera *Komagataeibacter*, *Acetobacter* and *Gluconobacter*, *Lactobacillus* and *Oenococcus*, while the commonly detected yeasts are belong to *Saccharomyces*, *Brettanomyces*, *Zygosaccharomyces*, *Pichia* and *Candida* (Wang et al., 2022b; Andreson et al., 2023). Through the complicated microbial interactions, abundant of metabolites are produced. These metabolites not only interfere with microbial succession during fermentation, its presence also directly influence the quality and health benefit of kombucha (Wang et al., 2022c).

In this study, we systematically investigated microbial community succession and metabolite changes in a back slopping kombucha fermentation process using high-throughput long-read amplicon sequencing and ultraperformance liquid chromatography–mass spectrometry (UPLC–MS), respectively. Unprecedented high sampling frequency of 2 days interval was performed. At the culmination of the study, correlations between these datasets were elucidated using canonical correspondence analysis (CCA). CCA is a multivariate statistical method used to understand the relationship between two sets of variables. Here, CCA will provide insights into how specific microbial populations might be influencing or being influenced by the production or consumption of metabolites during kombucha fermentation. This could help in understanding the fermentation dynamics and optimizing the process for desired characteristics.

## Materials and methods

### Preparation of kombucha

Kombucha samples were prepared following a back slopping fermentation method, to imitate a home-brewed kombucha fermentation. The original kombucha fermentation broth was obtained commercially from Taobao, an online business platform,^1^ in October 2021. Hereafter, it is named as the mother liquor. Black tea (10 g) was boiled in 1.4 L water for 10 min. After strained out the tea leaves, 70 g sucrose was added to prepare a sweetened tea broth. The tea broth was then divided evenly into 7 portions using sterilized 250 mL conical flasks. After cooling, 4 mL of mother liquor was spiked into each of the 200 mL sugared tea broth and left for fermentation at 28 ± 2°C. Sampling was carried out every two days interval for a period of 12 days fermentation, right before the onset of biofilm formation. The sample preparation and fermentation process were triplicated.

### Microbiota identification

(a) DNA extraction and amplification

Fermented samples were centrifuged at 12,000 *g* for 10 min, and pellet was collected for microbial DNA extraction using Bacteria Gen DNA kit (KangWei, Beijing) and Fungi DNA Isolation Kit (Norgen Biotek, Canada), following the manufacturer’s recommended standard operating procedures.

The Universal primers of 27F (5′-AGRGTTYGATYM TGGCTCAG-3′) and 1492R (5′- RGYTACCTTGTTACGACTT-3′) (Mamouri et al., 2023) were used to amplify the bacterial 16S rDNA V4 + V5 region. On the other hand, the fungal internal transcribed spacer (ITS) region was amplified using universal primers of ITS1: (5′-TCCTCCGCTTATTGATATGC-3′) and ITS4 (5′-TCCGTAGGTGAACCTGCGG-3′) (Matei et al., 2018). The PCR buffer (20 μL) used was composed of 4 μL of 5X FastPfu Buffer, 2 μL of 2.5 mM dNTPs, two primers (5 μM) of 0.8 μL each, 0.4 μL of FastPfu Polymerase, 1μL of template DNA and 11 μL of Sterile ultra-pure water. PCR amplification were performed with the following conditions: 94^°^C for 5 min, 30 cycles of 94^°^C for 1 min, 55^°^C for 1 min, 72^°^C for 1 min and a final extension of 72^°^C for 5 min.

Barcoded FIC primers were synthesized based on the designated sequencing region. PCR products were cleaned and extracted using a 2% agarose gels and AxyPrep DNA Gel Extraction Kit (Axygen Biosciences, Union City, CA, U.S.), respectively, following the manufacturer’s protocols.

(b). Sequence analysis

DNA library was constructed with the Illumina Pair-End library. Library quality in terms of concentration and fragment size of the amplicons were analyzed using Bioanalyser 2100 (Agilent Technologies). The amplicon library was subjected to a 2 × 250 paired-end sequencing procedure on the Illumina platform (Shanghai BIOZERON Biotech. Co., Ltd). The raw reads were then deposited to the NCBI Sequence Read Archive (SRA) database. Purified PCR products were quantified by Qubit^®^3.0 (Life Invitrogen). Details of the analysis procedure was as follows:

Firstly, raw fastq files were demultiplexed using an in-house perl script with the following conditions: (i) The 250 bp reads were truncated when an average quality score of < 20 over a 10 bp sliding window was recorded, and truncated reads shorter than 50 bp were discarded. (ii) exact barcode matching, 2 nucleotides were mismatched by primer-matching, reads with ambiguous characters were eliminated. (iii) overlapping sequences longer than 10 bp were assembled. Those reads failed to be assembled were discarded.

De-replicated reads were then de-noised followed by chimera checked using RDP Gold Database. Operational Taxonomic Units (OTUs) clustering was carried out with 97% similarities and clustered OTUs were phylogenetically identified with Silva 108 release database for yeast, while bacterial identification was executed with SILVA Incremental Aligner (SINA) tool against the SILVA and Green genes databases (Wang et al., 2007; Pruesse et al., 2012; Quast et al., 2012).

To classify the representative sequences of each OTU with 97% similarity, RDP classifier Bayesian algorithm (version 2.2)^2^ was used. Rarefaction analysis based on Mothur v.1.21.1 was conducted to determine Chao, ACE, and Shannon diversity indices (Schloss et al., 2009). Based on the taxonomic information acquired, the community structure was statistically analyzed at the species level. The Microbiome Analyst platform^3^ was used for heatmap analysis (Chong et al., 2020). The high-throughput sequencing data were deposited in the NCBI Sequence Read Archive under BioProjects PRJNA1129343 and PRJNA1129329.

### Untargeted metabolomic analysis

Untargeted metabolomic analysis was performed using an UHPLC-MS/MS system equipped with an Orbitrap Q Exactive™ HF mass spectrometer (Thermo Fisher, Germany). Kombucha sample (5 μL) was injected into a Hypesil Gold column (100 × 2.1 mm, 1.9 μm), running at a flow rate of 0.2 mL/min, at 35^°^C. The mobile phase gradient program with 0.1% formic acid in water (phase A) and 0.1% formic acid in acetonitrile (phase B) was as follows: 2% B, 1.5 min; 2–100% B, 12.0 min; 100% B, 14.0 min; 100–2% B, 14.1 min; 2% B, 17 min. The mass spectrometer was operated in positive/negative polarity mode with spray voltage of 3.2 kV, while the capillary temperature, sheath gas flow rate, and aux gas flow rate were controlled at 320°C, 40 arb, and 10 arb, respectively (Arri and Dragsted, 2013).

Kombucha liquid samples harvested at different fermentation days (Day 0, 2, 4, 6, 8, 10, and 12) were treated before analysis. Briefly, an aliquot of 100 μL sample was extracted with 1 ml of 80% methanol and 1 ml of 0.1% formic acid. The sample mixture was then subjected to vortex missing for 5 min before being centrifuged at 15,000 *g* for 20 min at 4^°^C. An aliquot of 600 μL of the supernatant was then dried in a vacuum concentrator and reconstituted in 100 μL of 50% methanol before being filtered into an auto-sampler vial. Sample injections were carried out at a random order. A quality control QC sample was prepared by pooling 10 μL from each sample. To ensure a constant analytical condition, the QC sample and blank samples were periodically injected throughout the analysis (Want et al., 2006; Pan et al., 2022).

Compound Discoverer 3.1 (CD3.1, Thermo Fisher) was used to perform alignment, identification, and quantitation of analyte peak. The parameters were set at 0.2 min of retention time tolerance; 5 ppm of actual mass tolerance; 30% of signal intensity tolerance; signal/noise ratio of 3; and 100,000 of minimum intensity. Sum of all peak intensities were normalized to the total spectral intensity. The normalized data was used to predict the molecular formula based on additive ions, molecular ion peaks and fragment ions. Identification was performed by peak matching using mzCloud,^4^ mzVault and MassList database.

The full list of compounds identified can be found in the Supplementary Dataset attached. Relative abundance changes of the following compounds as a function of fermentation time were determined:

1. Total sugar compounds.
2. Total nitrogenous compounds.
3. Total organic acid compounds
4. Caffeine.
5. Specific vitamins (i.e., Riboflavin, Pantothenic acid, Biotin, 4-oxoretinol).
6. Specific toxic biogenic amines (i.e., Tyramin, Spermine, N-(9-oxodecyl) acetamide, N-(4-morpholinophenyl)-4-(1H-pyrazol-1-yl) benzamide).

### Ethanol concentration and pH values

Ethanol concentrations were analyzed using enzymatic kits from Biosentec (Auzeville-Tolosane, France). The pH values of all samples were measured using a pH meter (Brand: ChuangYingHuanBao, Model BPH-252, Chengdu, China).

### Statistical analysis

IBM SPSS Statistics version 22 (SPSS, Chicago) was used for data analysis. Data was summarized by mean and standard deviation, assessed using analysis of variance (ANOVA) and Dunnett’s test for multiple comparisons. All statistical analysis was done with a 95% confidence level. The relative abundances changes in the bacterial and fungal community along fermentation period were classified by hierarchical cluster analysis and visualized by heatmap. Association of the dynamic changes in metabolites with the microorganism’s composition succession was assessed by canonical correspondence analysis (CCA). The CCA result plot uses dots to represent kombucha samples harvested at different fermentation days, and the arrows emanating from the origin representing different metabolite. It is worth noting that the length of the arrow represents the strength of the effect on the metabolite in association with the change in the microbial community, meaning to say the longer the arrow, the greater the effect on the metabolite. On the other hand, the Angle between the arrow and the axis represents the correlation between the metabolic substance and the axis, meaning the smaller the angle, the higher the correlation.

## Results and discussion

### Dynamic shift of bacteria and fungi community

As tabulated in Table 1, a total of 253,396 bacteria sequences were obtained from the 21 kombucha tea samples. The sequences were clustered into 398 OTUs with an average read length of 1371 bp and average Good’s Coverage index of 92.57%. Table 1 shows that the richness and evenness of kombucha tea bacterial community generally increased from Day 0 through Day 8, dropped drastically on Day 10 and Day 12. However, ACE and Chao1 indexes divided the fermentation period into three stages, within which an increased number of observed OTUs from a trough was observed, i.e., between Day 0–2, Day 4–8 and Day 10–12. This indicates that three distinct successive growth development of kombucha microbiota were evident, each with an increased number of bacterial species estimated. When the bacterial community species richness and evenness increases, the bacterial community diversity increases, which can be further elucidated by both the Shannon and Simpson indices. The higher the Shannon index, the more diverse the bacteria species in the kombucha tea. However, Simpson index is counterintuitive, where the higher the value the lower the diversity evenness. Thus, bacteria community structure observed specifically between Day 4 and Day 8 was showing relatively higher diversity (more richness and more evenness) than those observed in Stage 1 and Stage 3. Similarity in the microbial structure developed was further studied using Hierarchical cluster analysis.

**TABLE 1:** Alpha indexes of bacterial and fungal communities in kombucha tea during a 12-day fermentation period.

	Reads	Richness	Evenness	ACE	Chao1	Shannon	Simpson	Good’s Coverage
**Bacterial community**								
**Total Sequences (252,396); Average length (1371 bp); 398 OTUs.**								
KT0	10891 (717)^ab^	581 (53)^a^	0.45 (0.01)^b^	1006 (43)^a^	931 (21)^a^	4.13 (0.12)^b^	0.26 (0.01)^d^	0.97 (0.00)^c^
KT2	12283 (2431)^ab^	743 (36)^a^	0.37 (0.02)^a^	2824 (341)^a^	2634 (378)^b^	3.57 (0.19)^a^	0.32 (0.04)^e^	0.96 (0.01)^c^
KT4	13623 (1187)^b^	1388 (60)^c^	0.79 (0.00)^d^	1603 (61)^a^	1575 (72)^ab^	8.24 (0.05)^e^	0.02 (0.00)^a^	0.98 (0.00)^c^
KT6	11618 (1457)^ab^	3114 (302)^d^	0.89 (0.02)^e^	4423 (428)^b^	4322 (349)^c^	10.34 (0.36)^f^	0.01 (0.00)^a^	0.89 (0.02)^a^
KT8	9338 (911)^a^	4240 (241)^e^	0.95 (0.01)^f^	7315 (1848)^c^	6689 (1513)^d^	11.39 (0.13)^g^	0.00 (0.00)^a^	0.76 (0.07)^b^
KT10	10894 (364)^ab^	901 (53)^ab^	0.57 (0.02)^c^	1356 (64)^a^	1259 (66)^ab^	5.56 (0.27)^d^	0.09 (0.02)^b^	0.97 (0.00)^c^
KT12	13426 (1739)^b^	1090 (81)^b^	0.45 (0.01)^b^	2408 (198)^a^	2169 (126)^ab^	4.49 (0.02)^c^	0.20 (0.01)^c^	0.95 (0.00)^c^
**Fungal community**								
**Total sequence (288,605); Average length (609 bp); 111 OTUs.**								
KT0	9465 (2160)^A^	424 (15)^B^	0.46 (0.04)^B^	849 (51)^AB^	718 (5)^B^	4.00 (0.37)^B^	0.14 (0.03)^B^	0.97 (0.01)^A^
KT2	10190 (728)^A^	367 (10)^B^	0.43 (0.00)^B^	788 (31)^AB^	682 (29)^B^	3.68 (0.05)^B^	0.17 (0.01)^BC^	0.98 (0.01)^AB^
KT4	10596 (1525)^A^	436 (37)^B^	0.44 (0.04)^B^	601 (28)^AB^	566 (16)A^B^	3.86 (0.37)^B^	0.19 (0.04)^BC^	0.98 (0.01)^AB^
KT6	9519 (686)^A^	700 (192)^C^	0.61 (0.05)^D^	995 (438)^B^	895 (366)^B^	5.77 (0.52)^D^	0.08 (0.04)^A^	0.97 (0.01)^A^
KT8	7460 (360)^A^	552 (127)^C^	0.55 (0.02)^C^	847 (353)^AB^	751 (246)^B^	4.98 (0.34)^C^	0.14 (0.02)^B^	0.97 (0.01)^A^
KT10	20213 (4290)^B^	162 (32)^A^	0.14 (0.01)^A^	354 (121)^A^	291 (97)^A^	1.03 (0.11)^A^	0.77 (0.03)^D^	0.99 (0.01)^B^
KT12	11909 (672)^A^	386 (27)^B^	0.39 (0.00)^B^	855 (58)^AB^	781 (93)^B^	3.39 (0.05)^B^	0.22 (0.01)^C^	0.98 (0.01)^AB^

All values reported are mean values of triplicates and values in parentheses are standard deviation of the three measurements. Means with similar superscript letter (lowercase for bacterial community; uppercase for fungal community) within the same column are not significantly different at 0.05% probability. Sample codes of KT0 until KT12 indicate kombucha tea samples fermented by 0 through 12 days. Raw data are available at Supplementary Tables 1, 2.

**FIGURE 1:**
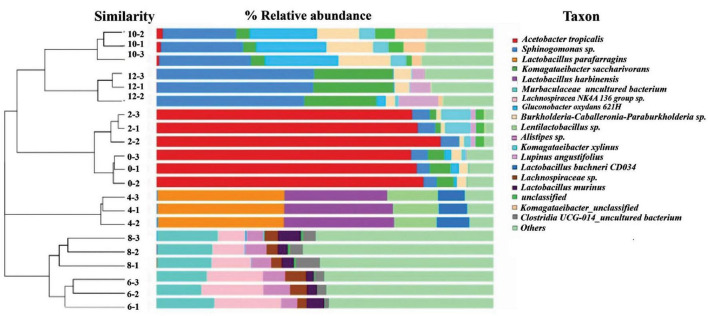
Hierarchical cluster analysis and relative composition of bacterial communities of kombucha tea samples throughout a 12-day fermentation. Number between the dendogram and heatmap represents sampling day and its replications (e.g., 10–2 indicates Day 10 and second replicate). Raw data is available at Supplementary Table 4.

Based on the dendrogram showed in Figure 1, succession of bacterial communities throughout the 12 days fermentations can be classified into three main clusters based on the bacterial community structure detected. The first cluster involved samples analysed on Day 0, Day 2, Day 10 and Day 12, which reveals that the bacterial community structure developed at the beginning and the late stage of kombucha fermentation are quite similar. The second cluster was mainly made up of Day 4 samples, which highly indicates Day 4 could be a transition period where the bacterial community showed a distinguished makeover, starting to accommodate a more diversify bacteria community. On the other hand, Cluster 3 involved samples analysed on Day 6 and Day 8, which showed the highest structure richness and evenness.

Figure 1 also shows the kombucha’s microbial relative composition throughout the 12 days of fermentation, and detailed composition data could be found in Supplementary Table 4. In the early stage of fermentation (Day 0, Day 2), it is noted that the dominant bacteria were *Acetobacter tropicalis* (77.5%, 79.5%), followed by *Komagataeibacter saccharivorans* (5.5%, 1.2%), *Sphingomonas* spp. (4.2%, 5.21%) and *Komagataeibacter xylinus* (0.4%, 6.0%). Both *Komagataeibacter saccharivorans* and *Komagataeibactor xylinus* are found to be capable of synthesizing cellulose (Gopu and Govindan, 2018; Sknepnek et al., 2020; Nyhan et al., 2022). On Day 4, lactic acid bacteria (LAB) were found to be the dominating ones, the specific strains detected includes *Lactobacillus parafarragins* (37.5%), *Lactobacillus harbinensis* (32.0%), *Lentilactobacillus* sp. (13.7%) and others microbial species accounting for 16.8% of the total OTUs. Kombucha fermentation proceed to Day 6 and Day 8 with the diversity of the bacterial community increased significantly, and major bacteria includes *Muribaculaceae* (15.1–16.8%) and *Lachnospiraceae* (9.8–12.7%). On Day 10, the dominant bacteria were *Gluconobacter oxydans* (21.0%), *Sphingomonas* (24.5%) and *Burkholderia-Caballeronia-Paraburkholderia* (14.0%). On Day 12, *Sphingomonas* spp. *(45.7%)* and *Komagataeibacter* spp. (23.4%) thrived and grow vigorously. From Figure 1, it is worth noting that though the microbial community structure was detected to be quite similar for the early (Day 0–2) and late (Day10–12) stage of kombucha fermentation, the relative abundance among species is vastly different, whereby the dominant bacterium in the early stage was found to be *Acetobacter* spp., while *Sphingomonas* spp. was the major bacterium in the later stage.

**FIGURE 2:**
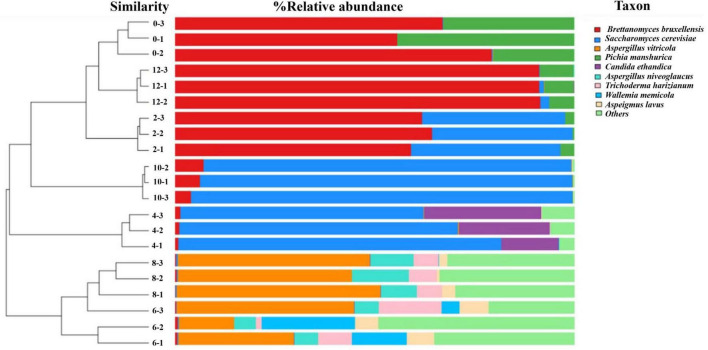
Hierarchical cluster analysis and relative composition of fungal communities of kombucha tea samples throughout a 12-day fermentation. Number between the dendogram and heatmap represents sampling day and its replications (e.g., 0–3 indicates Day 0 and third replicate). Raw data is available at Supplementary Table 3.

In this study, a total of 288,605 fungal gene sequences were generated for fungal community analysis. The sequences were clustered into 111 OTUs with an average length of 609 bp and an average Good’s Coverage index of 97.71%. Results in Table 1 show that both the fungal community richness and evenness increased progressively from Day 0 through Day 6, and drop progressively from Day 6 through Day 10, and lastly increase marginally from Day 10 to Day 12. Similar trend is observed on indices of ACE, Chao1, and Shannon. From the hierarchical analysis results (Figure 2), it is showed that fungal community structure of Day 0, 2, 10 and 12 are clustered closely, but it was found significantly different from those of Sample Day 4, 6 and 8. Again, Day 4 fungal community structure is seen to be classified as a stand-alone cluster that signified the transition period, where a revamped fungal community structure was seen thereafter and through Day 6 and Day 8. From the relative composition showed in Figure 2, the pioneer fungal species detected on Day 0 were *Brettanomyces bruxellensis* (67.3%) and *Pichia manshurica* (32.5%), with traces of *Saccharomyces cerevisiae* (0.2%). On Day 2, *Brettanomyces bruxellensis* remained as the dominant fungi and accounting for 61.8% of all OTUs, while *Saccharomyces cerevisiae* (36.2%) grew very fast and *Pichia manshurica* (2.1%) decreased. On Day 4, *Saccharomyces cerevisiae* continued to grow (70.6%) in the presence of *Candida ethanolica* (22.1%) and *Pichia kudriavzevii* (5.0%). On Day 6 and Day 8, a different fungal community structure was detected. *Aspergillus* spp. was found to flourish steadily, where relative abundance of *Aspergillus vitricola increased from 29.2% to 49.3%* and *Aspergillus niveoglaucus* increased from 5.8% to 11.4%. As seen in Figure 2, *Saccharomycess cerevisiae* (93.7%) and *Brettanomyces bruxellensis* (91.3%) are the predominant fungi grew on Day 10 and Day 12, respectively. *Brettanomyces bruxellensis* is one of the potent wine contaminants because it produces phenolic off-flavors such as 4-ethylphenol (Branco et al., 2021).

Upon cross-referencing our findings with existing literature, we identified several predominant bacteria and fungi genera typically associated with kombucha fermentation. These include *Komagateibacter, Gluconobacter, Lactobacillus, Oenococcus, Pseudomonas, Burkholderia, Ralstonia, Halomonas*, and *Sphingomonas* among bacteria, and *Saccharomyces, Dekkera/Brettanomyces, Zygosaccharomyces, Pichia, Torulaspora, Candida, Schizosaccharomyces, Botryotrichum*, and *Monascus* among fungi (Reva et al., 2015; Coton et al., 2017; Villarreal-Soto et al., 2020; Andreson et al., 2022; Meng et al., 2024).

Interestingly, *Aspergillus*, *Pseudomonas* and *Candida* are found present in kombucha. It is worth noting that some species of this bacteria could be harmful if ingested (Nyhan et al., 2022). Contaminated mother liquor, poor fermentation conditions and cleanliness of the brewing environment could account for these observations. Therefore, it’s important to ensure that kombucha is brewed under sanitary conditions and that only appropriate ingredients are used to minimize the risk of pathogenic contamination. Additionally, the acidic nature of kombucha and competition among microorganisms during fermentation can help inhibit the growth of pathogens (Nyhan et al., 2022).

Our analysis unveiled dynamic fluctuations in the microbial community structure throughout the 12-day sampling period. These shifts are indicative of the intricate microbial succession process inherent to kombucha fermentation, where the composition of microorganisms evolves in response to changes in the brewing medium. Factors such as the introduction of new nutrients (i.e., tea components, sugar, by-products of fermentation), shifts in pH levels, and alterations in environmental conditions contribute to this dynamic microbial community assembly. While geographical origin and the initial microbial composition of the mother culture may play a role, the primary driver of these fluctuations is the ongoing fermentation process itself. Each stage of fermentation presents unique ecological niches that select for specific microorganisms, resulting in a dynamic and evolving community over time (Barbosa et al., 2021).

It’s worth noting that these shifts in microbial composition can significantly impact the quality and safety of the final kombucha product. The presence or absence of various genera directly influences the production of beneficial or potentially harmful metabolites, underscoring the importance of understanding and monitoring microbial dynamics throughout the fermentation process.

### Metabolites analysis

**FIGURE 3:**
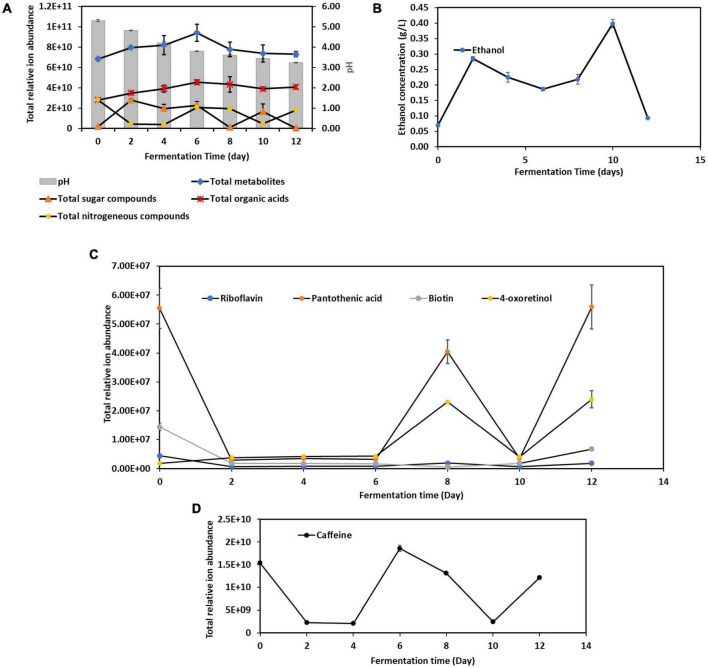
Dynamic changes of metabolites during the 12-day fermentation of kombucha tea. **(A)** pH, total metabolites, sugar compounds, organic acids and nitrogenous compounds; **(B)** ethanol concentration; **(C)** vitamins compounds; **(D)** caffeine. Raw data is available at Supplementary Tables 6, 8–11.

A total of 486 metabolites produced during the 12-Day kombucha fermentation were detected by UHPLC-MS/MS (Supplementary Table 5). The variations in the overall metabolites’ composition are depending on the initial microbial structure of the SCOBY or liquid starter and those developed throughout the fermentation process (Barbosa et al., 2021). As seen in Figure 3A, the total number of metabolites identified increased from Day 0 through Day 6, and progressively decreased from Day 6 through Day 12. Interestingly, the dynamic changes in the total sugar compounds and the nitrogenous compounds are in a countervailing trend, where the two lines (Figure 3A) are noted to be pulling in opposite directions, meaning an increase in nitrogenous compounds will be accompanied by a decrease in sugar compounds, and vice versa.

Apart from these, Figure 3A also depicts that pH value of the kombucha tea broth decreased progressively from 5.30 to 3.23 with fermentation time, which in line with result reported in Wang et al. (2023). This is due to the activity of both acetic acid bacteria and lactic acid bacteria, who metabolize ethanol produced by yeasts, into acetic acid and lactic acid respectively, which lowers the pH. In addition, there are other organic acids being produced by other microorganisms, with gluconic acids, succinic acids, gallic acids being the predominant organic acids in the final product (Supplementary Table 7). It is worth noted that succinic acid is known as a “natural” antibiotic and gallic acid has been reported to have good antioxidant and anti-inflammatory properties (Dexlin et al., 2024). This acidic environment is crucial because it helps to inhibit the growth of contaminant microorganisms, including pathogens, ensuring the safety of the kombucha (Nyhan et al., 2022).

Meanwhile, the ethanol concentration (Figure 3B) increased quickly during the first two days of fermentation. Between Day 2 and Day 10, a trough is noticed on Day 6, and ethanol content dropped again after Day 10. The initial rise in ethanol was attributed to yeast actively fermented the available sucrose into ethanol and carbon dioxide. However, as the fermentation processes, the dynamic changes in nutrient availability, along with the inhibitory effects of acids and antimicrobial compounds, shape the microbial community, leading to the observed shifts in dominance (Utoiu et al., 2018; Tu et al., 2019). As discussed previously, *Aspergillus* spp. was seen outcompete *Saccharomyces cerevisiae* in the mid stage, however soon after yeasts resuscitated and fluorished. These account for the ethanol concentration fluctuations in kombucha fermentation.

From Figure 3C, it is found that kombucha tea may be a good source of pantothenic acid (vitamin B5) (Bishop et al., 2022). Pantothenic acid is required for synthesizing of coenzyme A, that is essential for metabolizing fatty acid and energy production. In addition, 4-oxoretinol, a biologically active retinoid (Achkar et al., 1996) also detected to be present in Kombucha. However, their relative ion abundance fluctuated throughout the fermentation process. The fluctuations in 4-oxoretinol and pantothenic acid levels during kombucha fermentation are likely due to complex interactions between microbial metabolism, nutrient availability, and environmental factors. Obviously, the drastic drop of pantothenic acid by Day 2 indicates that it could have been utilized to support the growth of certain microbes. The rise in levels by Day 8 suggests active microbial synthesis, while the drop by Day 10 indicates increased utilization or transformation by the microbial community. The subsequent rise by Day 12 may reflect a secondary phase of microbial activity or changes in the microbial community dynamics.

Caffeine is a naturally occurring xanthine alkaloid in tea. Basically, the total ion abundance of caffeine (Figure 3D) fluctuated in the same manner as the total nitrogenous compounds (Figure 3A), but in a countervailing trend to ethanol content, which could be attributed to the fact that caffeine serves as a nitrogen source for yeast (specifically *Saccharomyces* spp.) for metabolic processes and new cells building (Crum et al., 2016).

### Biogenic amines

Biogenic amines (BAs) represent a significant risk factor in traditional fermented foods due to their potential vasoactive, psychoactive, and toxicological effects (Vijayaraghavan et al., 2000). These compounds, including tyramine, histamine, putrescine, cadaverine, phenylethylamine, and tryptamine, possess biological activity at low relative molecular weights. While trace amounts of BAs play specific physiological roles, excessive ingestion from various sources can pose serious health risks (Costa et al., 2018).

**FIGURE 4:**
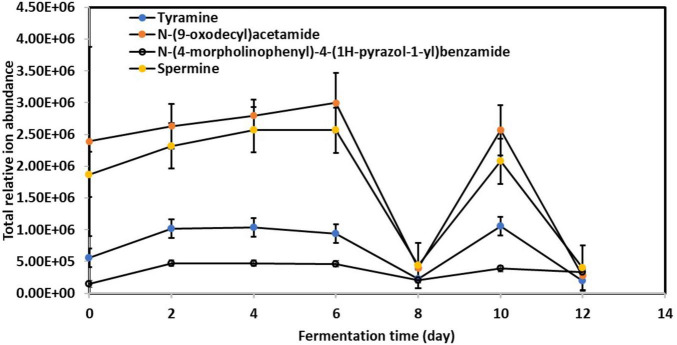
Relative changes in toxic biogenic amines content in kombucha samples throughout the 12-day fermentation. Raw data is available at Supplementary Table 12.

Figure 4 illustrates the significant abundance of specific BAs, including tyramine, spermine, N-(9-oxodecyl)acetamide, and N-(4-morpholinophenyl)-4-(1H-pyrazol-1-yl)benzamide, in the fermented kombucha tea samples. Remarkably, these BAs exhibited a consistent pattern of behavior throughout the fermentation process. Initially, their abundance increased progressively from Day 0 to Day 6, experienced a drastic drop on Day 8, increased again on Day 10, and subsequently decreased by Day 12.

The observed fluctuations in BAs content can be attributed to the complex interplay of microbial activity during kombucha fermentation. Research by Ladero et al. (2012) highlights the role of certain microorganisms, particularly lactic acid bacteria, in synthesizing BAs through the decarboxylation of amino acids. Specifically, the progressive increase in BAs abundance from Day 0 to Day 6 (Figure 4) may correlate with the predominant presence of microorganisms such as *Lactobacillus parafarraginis, Lactobacillus harbinensis*, and *Lentilactobacillus*, which are the predominant microorganisms grew on Day 4. Apart from this, Figure 1 also depicts high relative abundance of other microorganisms (e.g., *Muribaculaceae* sp., *Aspergillus* sp., etc.) grew on Day 6, which could also account for the high relative abundance of BAs detected. Conversely, the subsequent drop in BAs levels after Day 6 could result from the metabolic activity of other microorganisms capable of metabolizing these compounds, such as *Lactobacillus* spp. and *Saccharomyces* sp. which flourished during mid stage of kombucha fermentation (Figures 1, 2). This observation is supported by Li et al. (2018) and Sun et al. (2022).

It’s crucial to recognize that the dynamic changes in BAs content over the fermentation period reflect the ongoing production and consumption of these compounds by different microbial strains. Therefore, understanding the microbial ecology and metabolic pathways involved in BAs synthesis and degradation is essential for managing their levels in kombucha tea. Moving forward, further investigation into the specific microbial strains responsible for BAs production and degradation, as well as the factors influencing their activity, will be instrumental in developing strategies to mitigate the health risks associated with BAs in fermented foods like kombucha tea.

### Canonical Correspondence Analysis (CCA)

**FIGURE 5:**
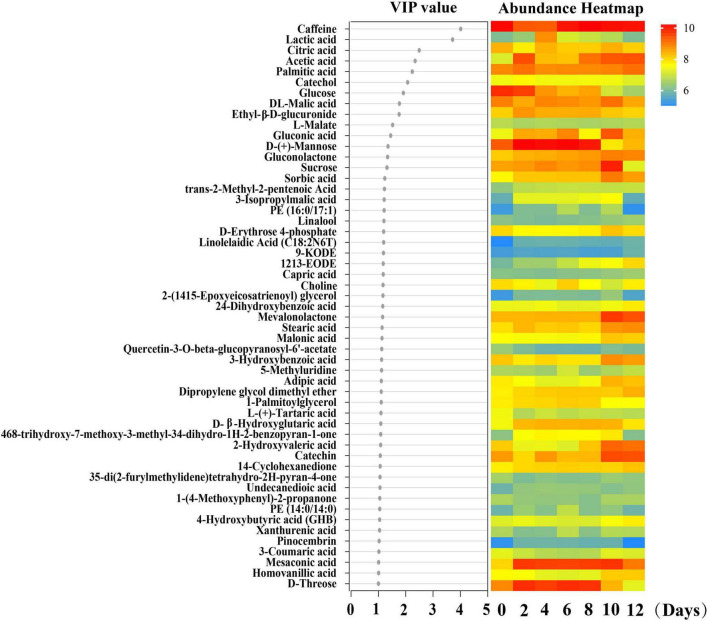
PLS-DA analysis of the major differential metabolites detected during kombucha fermentation. VIP value of the major differential metabolites is shown and the color scale in the heatmap represents the relative concentration of the metabolites at different sampling time.

Before delving into the correlation between species abundances and metabolites throughout the kombucha fermentation period, Partial Least Squares Discriminant Analysis (PLS-DA) was initially conducted to identify the differential metabolites produced over the fermentation days. From this analysis, a total of 15 differential metabolites with VIP (Variable Importance in Projection) scores exceeding 1.3 were selected to proceed with the correlation study. These metabolites included caffeine, lactic acid, citric acid, acetic acid, palmitic acid, catechol, glucose, DL-malic acid, ethyl-β-D-glucuronide, L-malate, gluconic acid, D-(+)-mannose, gluconolactone, sucrose, and sorbic acid (Figure 5).

**FIGURE 6:**
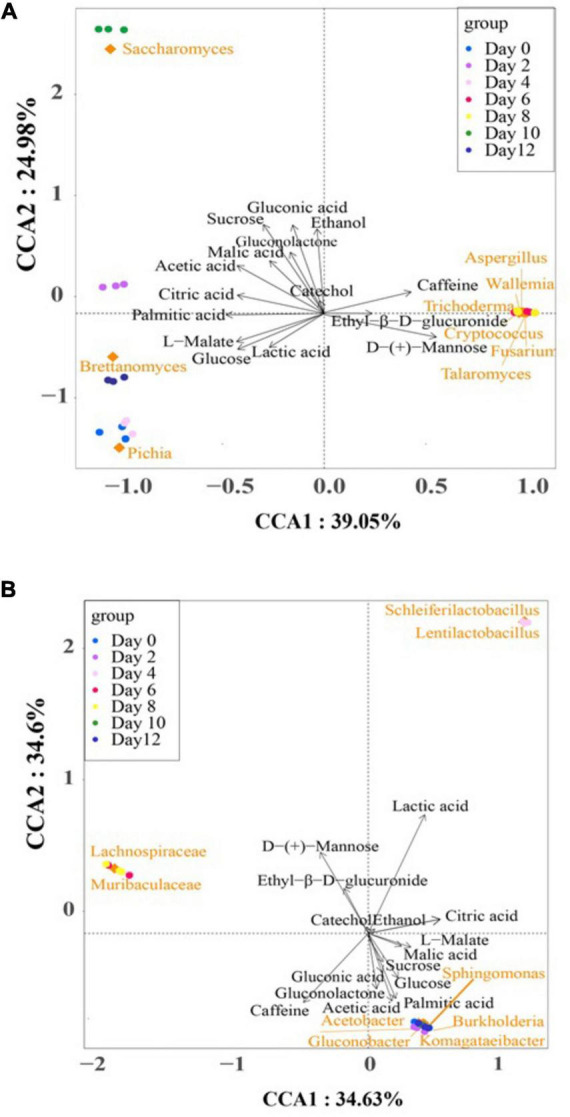
Canonical correspondence analysis of dominant microorganisms, fermentation time, and the major differential metabolites during kombucha fermentation. **(A)** is for fungus and **(B)** is for bacterium. Arrows represent different metabolites. Blocks represent dominant microorganisms. Circles represent different fermentation time.

Figure 6 illustrates the Canonical Correspondence Analysis (CCA) map, providing a simultaneous visualization of the correlations between microbial communities and the selected differential metabolites across the kombucha fermentation timeline. The two axes of the CCA map collectively explained 69.23% and 64.03% of the total variance in bacterial and fungal communities’ differentiation, respectively, indicating significant correlations between microbial community dynamics and metabolite production.

In the bacteria CCA map (Figure 6A), distinct clusters and associations were observed at different stages of fermentation. Notably, *Komagataeibacter* spp., *Sphingomonas* spp., *Acetobacer* spp., *Gluconobacter* spp., and *Burkholderia* spp. were closely clustered and positively associated with acetic acid, palmitic acid, gluconolactone, glucose, gluconic acid, and sucrose, particularly during the early (Day 0–2) and late (Day 10–12) stages of fermentation. Conversely, lactic acid exhibited a strong association with *Scheleiferilactobacillus* spp. and *Lentilactobacillus* spp. on Day 4, while the abundance of *Lachnospiraceae* spp. and *Muribaculaceae* spp. on Day 6 and 8 positively correlated with D-(+)-mannose and ethyl-β-D-glucuronide. According to Wang et al. (2024), total acid and acetic acid levels reduced substantially with the addition of lactic acid bacteria, specifically *L. plantarum*, into kombucha. In addition, pivotal volatile compounds also increased and enriched a fruity and flora aroma of the kombucha.

In the fungus CCA map (Figure 6B), distinct associations between fungal species and differential metabolites were observed throughout the fermentation period. *Pichia* spp., *Brettanomyces* spp., and *Saccharomyces* spp. were associated with several metabolites, while *Aspergillus* spp., *Wallemia* spp., *Trichoderma* spp., *Cryptococcus* spp., *Fusarium* spp., and *Talaromyces* spp. were attributed to the presence of caffeine, ethyl-β-D-glucuronide, and D-(+)-mannose on Day 6 and Day 8. Notably, these later groups represent “contaminant” microorganisms that may adversely affect the quality of kombucha.

Ethanol was identified as the dominant alcoholic compound in the fermentation broth, with *Saccharomyces* spp. playing a crucial role in alcohol production, particularly on Day 10. This observation suggests the potential for regulating ethanol content in kombucha by shortening the fermentation period to less than 10 days. Such findings align with literature reports recommending 6–7 days of fermentation to achieve a sensorily acceptable kombucha beverage with minimal alcohol and vinegar flavor notes (Li et al., 2022).

### Summary of the correlations between microbiota succession and the metabolites dynamic changes

**FIGURE 7:**
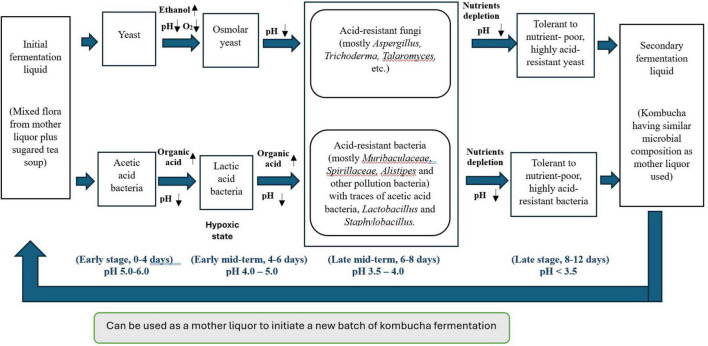
Schematic overview of the correlations between microbiota succession and metabolic ecology in different kombucha fermentation stages.

To elucidate the intricate dynamics of microbial community succession and metabolite changes throughout the fermentation of traditional kombucha, we have developed a schematic diagram (Figure 7) based on a 12-day fermentation period.

During the initial days of kombucha fermentation (Days 0–4), the primary microorganisms involved are yeasts, notably *Brettanomyces bruxellensis*, *Saccharomyces cerevisiae* and *Pichia manshurica*. These yeasts begin the fermentation process by converting the available sugars into ethanol and carbon dioxide. Concurrently, *Acetobacter tropicalis* start to grow and converting sugars into acetic acid and contributing to an initial decrease in pH. The presence of lactic acid bacteria (LAB) on Day 4 of kombucha fermentation is not uncommon. If the kombucha culture has been used in multiple fermentation cycles, LAB from previous batches could persist in the SCOBY or liquid starter. In addition, the tea leaves, sugar and even the water used can introduce LAB into the fermentation process. LAB can convert sugars into lactic acid, which further lowers the pH of kombucha. This acidic environment inhibits the growth of pathogenic microorganisms, enhancing the safety of the drink and sets the stage for subsequent microbial activities. Apart from being a probiotic, through the production of lactic acid and other metabolites, LAB contributes to the complex, tangy flavor profile of kombucha. This can complement the acetic acid produced by acetic acid bacteria.

The diverse bacterial community and the growth of Aspergillus spp. observed from Day 6 to Day 8 in kombucha are the result of the natural progression of microbial succession, nutrient availability, and environmental conditions favoring acid-tolerant organisms. This highlights the complexity and dynamic nature of kombucha fermentation.

By Days 8 to 12, the kombucha environment is characterized by a low pH (around 3.5) and more complex nutrient availability. This stage sees the dominance of *Saccharomyces cerevisiae* (Day 10) and *Brettanomyces bruxellensis* (Day 12), a yeast known for its tolerance to both acidic conditions and ethanol. Both *Saccharomyces* and *Brettanomyces* can metabolize complex sugars and contribute unique flavor compounds, which add to the complexity of the final kombucha’s taste. Acetic acid bacteria, such as *Komagataeibacter* spp. and *Gluconobacter* spp., continue to thrive, converting any remaining ethanol into acetic acid and producing bacterial cellulose, which helps form the structure of the SCOBY. *Sphingomonas* spp. also persist due to their versatile metabolism and ability to survive in the low pH environment.

Throughout the entire fermentation process, the microbial diversity and interactions create a dynamic ecosystem. Early stages are dominated by yeasts, acetic acid bacteria and lactic acid bacteria, the mid stage sees a rise in diverse bacteria and fungi community, and the later stages are characterized by acid-tolerant yeasts and acetic acid bacteria. The pH gradually decreases due to the production of organic acids, stabilizing around 3.5. Nutrient availability shifts from simple sugars to more complex substrates, favoring different microorganisms at different stages. This succession illustrates the complex interplay of microbial activities that define the unique characteristics of kombucha.

## Conclusion

In summary, this study highlights the importance of integrating advanced analytical techniques with traditional fermentation practices to achieve optimal outcomes in kombucha production. Through canonical correspondence analysis, correlations between specific microbial populations and metabolites have been identified. By leveraging this knowledge and utilizing commercial starter cultures, producers can continue to refine and innovate fermentation processes to meet evolving consumer demands for safe, flavorful, and health-promoting kombucha tea products. However, due to the detection of harmful biogenic amines in the kombucha fermentation process, future research could focus on correlating the presence and quantity of harmful biogenic amines with specific microbial strains and verify its significance through functional studies by inoculating sterilized kombucha base with individual strains and measuring the production of biogenic amines.

## Data availability statement

The datasets presented in this study can be found in online repositories. The names of the repository/repositories and accession number(s) can be found in the article/Supplementary material.

## Author contributions

TL: Data curation, Formal analysis, Investigation, Methodology, Writing–original draft. X-RL: Data curation, Formal analysis, Writing–original draft. LF: Methodology, Writing–original draft. BZ: Funding acquisition, Writing–original draft. W-MZ: Investigation, Writing–original draft. J-JH: Investigation, Writing–original draft. LL: Methodology, Supervision, Writing–review and editing. NM: Supervision, Writing–review and editing. L-HC: Conceptualization, Supervision, Writing–review and editing.

## Footnotes

1
https://m.tb.cn/h.gQh6sox5Lxf2TvS?tk=WOY232ySPzbCZ8908

2
http://sourceforge.net/projects/rdp-classifier/

3
https://dev.microbiomeanalyst.ca

4
https://www.mzcloud.org/
